# Delayed Diagnosis of Coarctation of the Aorta in a Hypertensive Child: Challenges in Early Detection

**DOI:** 10.7759/cureus.85253

**Published:** 2025-06-02

**Authors:** Ryunosuke Hisatomi, Siddharth Mahajan, Ruikang Liu

**Affiliations:** 1 Family Medicine, LSU Health Shreveport, Shreveport, USA; 2 Cardiology, LSU Health Shreveport, Shreveport, USA

**Keywords:** blood pressure measurement, coarctation of the aorta, ct angiography, diagnostic delay, echocardiography, femoral pulse, pediatric hypertension

## Abstract

Coarctation of the aorta (CoA) remains a frequently underrecognized cause of secondary hypertension in children. We report a case of a preadolescent with persistent, treatment-resistant hypertension initially evaluated with normal echocardiograms and extensive workups, including renal, endocrine, and genetic testing. Importantly, lower extremity blood pressure and femoral pulse assessment were omitted during initial evaluations. After referral to pediatric cardiology, diminished femoral pulses were detected and a third echocardiogram revealed severe CoA, later confirmed by CT angiography. The patient underwent successful stent placement, resulting in blood pressure normalization and discontinuation of all antihypertensive medications. This case highlights the diagnostic pitfalls associated with incomplete physical examination and reliance on a single imaging modality. It reinforces the importance of guideline-based evaluation for pediatric hypertension, including routine assessment of femoral pulses and four-limb blood pressure measurements. In cases of persistent hypertension with high clinical suspicion despite inconclusive initial imaging, repeat echocardiography or advanced imaging should be considered to prevent diagnostic delays and optimize outcomes.

## Introduction

In contrast to adults, where primary hypertension is more common, secondary hypertension is the leading cause of elevated blood pressure in children, particularly those under 15 years of age [1]. The most common causes include renal parenchymal disease, renovascular abnormalities, and endocrine disorders. Although less common, cardiovascular anomalies such as coarctation of the aorta (CoA) should also be considered [2]. CoA is a congenital narrowing of the aorta that can result in upper body hypertension, left ventricular hypertrophy, and long-term complications including cerebrovascular events and heart failure [3]. Although CoA affects approximately four per 10,000 live births, it is frequently underdiagnosed because its clinical signs may be subtle and routine screening methods often fail to detect it [4]. Early recognition relies heavily on guideline-based physical examination, including lower extremity blood pressure measurement and femoral pulse palpation. While echocardiography is commonly used as an initial imaging modality, it has limited sensitivity in detecting CoA, particularly in older children. Prompt diagnosis and intervention are crucial to prevent serious complications and improve long-term outcomes.

## Case presentation

An 11-year-old girl presented to our tertiary care unit seeking a second opinion for persistent hypertension after multiple previous evaluations and unsuccessful treatment efforts. The patient had been initially diagnosed one year prior following an emergency department visit for a hypertensive emergency. Despite comprehensive evaluations and antihypertensive therapy, blood pressure remained uncontrolled. The patient had a history of hypertension and acne, with no known congenital heart disease, renal disorders, or metabolic conditions. Family history was unremarkable. The patient was adherent to antihypertensive medications, followed the Dietary Approaches to Stop Hypertension (DASH) diet, and engaged in daily physical activity. The patient’s height and weight were both around the 80th percentile for age and sex. Pubertal development was appropriate for age. At presentation, the patient was on a regimen of three different antihypertensive agents (amlodipine 5 mg, lisinopril 10 mg, and hydrochlorothiazide 25 mg), yet blood pressure remained persistently above the 99th percentile for age, sex, and height. The patient reported occasional headaches, epistaxis, and leg cramps.

Previous evaluations at other institutions included laboratory tests such as renin and aldosterone levels, 24-hour ambulatory blood pressure monitoring, renal ultrasound, echocardiography, and genetic testing for monogenic hypertension. Ambulatory monitoring confirmed stage 1 hypertension, while laboratory tests, renal ultrasound, and echocardiography were unremarkable. Genetic testing identified a variant of uncertain significance, which was deemed unrelated to the patient’s clinical presentation.

Repeated laboratory tests (Table 1) and echocardiography were unremarkable. The patient was referred to endocrinology, where adrenal and thyroid hormone levels, as well as autoimmune disease testing, were within normal limits. There was no evidence of pheochromocytoma, endocrine-related hypertension, or an autoimmune etiology. Due to complaints of snoring and daytime sleepiness, the patient was evaluated for obstructive sleep apnea (OSA). Polysomnography confirmed mild OSA. However, the apnea index was below the continuous positive airway pressure (CPAP) treatment threshold, and there was no evidence of excessive daytime sleepiness or significant nocturnal desaturation. Therefore, OSA was not considered a primary cause of her severe hypertension.

**Table 1:** Laboratory test results AST: aspartate aminotransferase; ALT: alanine transaminase; TSH: thyroid-stimulating hormone; ANA: antinuclear antibody; DHEA-SO_4_: dehydroepiandrosterone sulfate

Laboratory test	Result	Reference range	Unit
White blood cell (WBC) count	6.73	4.50-14.50	K/µL
Hemoglobin	10.9	11.5-15.5	g/dL
Platelet count	336	150-450	K/µL
Sodium	137	136-145	mmol/L
Potassium	3.9	3.5-5.1	mmol/L
Blood urea nitrogen (BUN)	11	5-18	mg/dL
Creatinine	0.66	0.50-1.40	mg/dL
Calcium	8.8	8.7-10.5	mg/dL
Total protein	7.6	6.0-8.4	g/dL
Albumin	3.4	3.2-4.7	g/dL
Total bilirubin	0.2	0.1-1.0	mg/dL
Alkaline phosphatase	294	141-460	U/L
AST	19	10-40	U/L
ALT	23	10-44	U/L
TSH	1.460	0.400-5.000	µIU/mL
Free T4	1.06	0.71-1.51	ng/dL
Cortisol	4.03	3.43-16.75	µg/dL
ANA screen	Negative	Negative	
Testosterone, free	<0.4	<2.7	pg/mL
DHEA-SO_4_	111	11-296	µg/dL
17-Hydroxyprogesterone	63	<100	ng/dL
Aldosterone	28.8	≤39.2	ng/dL

**Figure 1:**
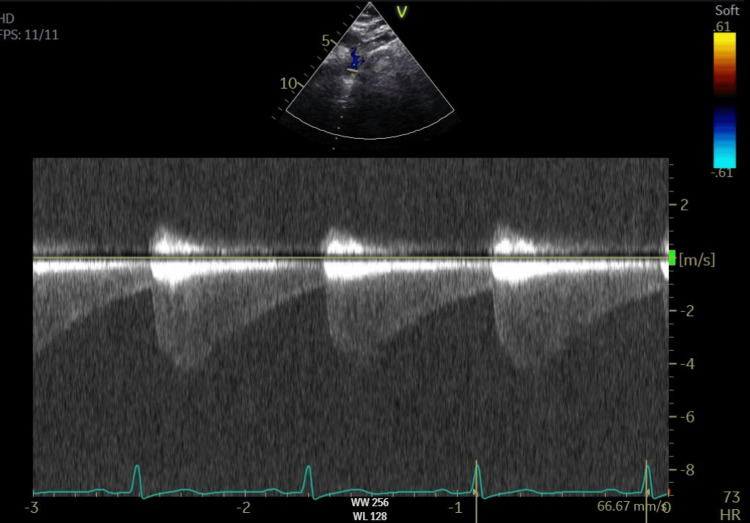
Repeat echo Suprasternal view of the aortic arch - continuous wave Doppler through the aortic isthmus with high-velocity systolic amplitude and continuous antegrade flow through diastole consistent with severe coarctation of the aorta. The peak pressure gradient was 78 mmHg, with a mean gradient of 40 mmHg.

**Figure 2:**
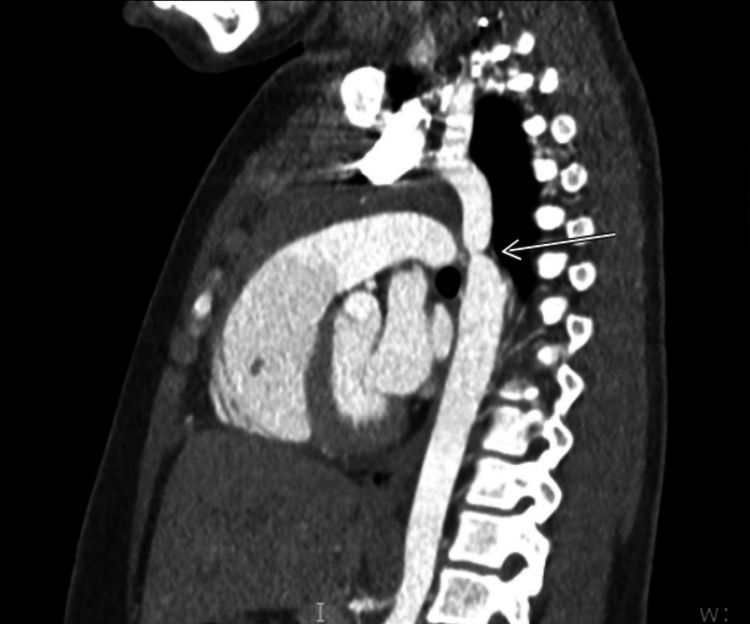
CT angiography The thoracic aorta shows narrowing of the proximal thoracic descending aorta measuring 10 mm in transverse diameter.

Despite multiple evaluations, lower extremity blood pressure measurement and pulse assessment were never performed. Given persistent hypertension despite pharmacologic and lifestyle interventions, the patient was referred to a pediatric cardiologist approximately one year after our initial evaluation. On examination, femoral pulses were diminished bilaterally on palpation, suggesting impaired distal perfusion. A third echocardiogram identified severe CoA with a peak gradient of 78 mmHg, which had previously been unrecognized (Figure 1). CT angiography confirmed a discrete narrowing of the descending thoracic aorta, measuring 10 mm in diameter (Figure 2).

**Figure 3:**
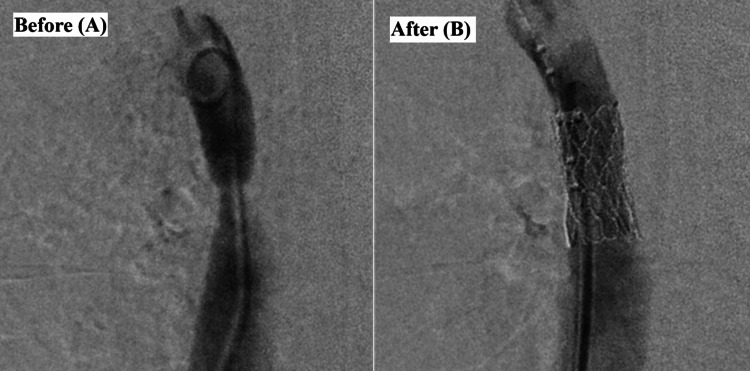
Cardiac catheterization Before (A) and after (B) transcatheter stent placement.

The patient was subsequently referred to cardiac surgery and underwent cardiac catheterization, during which a 14 mm × 2.8 cm covered Cheatham-Platinum (CP) stent was successfully placed to alleviate the aortic narrowing (Figure 3). The procedure was performed via right femoral arterial access using a 12 Fr sheath. The procedure was well tolerated, with no immediate complications. Post-procedural echocardiography confirmed the complete resolution of the coarctation, with a residual pressure gradient of 0 mmHg. Femoral pulses were easily palpable bilaterally after the procedure.

At three months postoperatively, the patient’s blood pressure remained well controlled, permitting a gradual tapering of antihypertensive therapy. By six months post-procedure, blood pressure remained stable, allowing for the discontinuation of all antihypertensive medications. Repeat echocardiography demonstrated no evidence of restenosis, and cardiovascular function remained stable. The patient will continue routine follow-up with pediatric cardiology, including periodic imaging to monitor for potential restenosis.

## Discussion

Secondary hypertension is the most common cause of hypertension in children, particularly those under 15 years of age. The most frequent etiologies include renal parenchymal disease, endocrine disorders, and renovascular abnormalities [1,2]. Although less common, cardiovascular causes such as CoA should not be overlooked. If undiagnosed, CoA can lead to significant morbidity, including left ventricular hypertrophy, heart failure, aortic aneurysm formation, and cerebrovascular events such as stroke or rupture of an intracranial aneurysm [3].

Recognizing CoA in older infants and children is particularly challenging due to the subtle nature of clinical signs and the frequent absence of overt symptoms. Therefore, a thorough medical history and physical examination are essential. Symptoms may include hypertension, along with signs of impaired circulation such as headaches, epistaxis, and leg discomfort during physical activity. Classic clinical findings include systolic hypertension in the upper extremities relative to the lower extremities and diminished or delayed femoral pulses (brachial-femoral delay) [3].

The American Academy of Pediatrics (AAP) hypertension guidelines emphasize the importance of incorporating lower extremity blood pressure measurement and femoral pulse assessment into routine evaluations. These assessments are recommended at the second visit for confirmed elevated blood pressure or stage 1 hypertension and at the first visit for stage 2 hypertension [5]. A study found that femoral pulses were absent in 40% of cases, while pedal pulses were absent in 77% [6]. Additionally, 97.9% of patients with CoA exhibited an upper limb blood pressure measurement at least 20 mmHg higher than that in the lower limbs [7]. Despite its high diagnostic sensitivity, lower limb blood pressure measurement was not performed in our case, which may have contributed to the delayed diagnosis. Similarly, lower extremity pulse assessment was documented only after the patient was evaluated by a cardiology specialist.

Unfortunately, our case is not an isolated occurrence. Another study reported that in a cohort of 38 patients aged 12-18 years diagnosed with CoA, 57.8% had not undergone lower extremity blood pressure measurement [7]. Similarly, routine lower limb blood pressure measurement was performed in only 30.3% of hospitalized children, with an additional 44.0% undergoing measurement only when CoA or other vascular diseases were suspected [8].

These findings in our case mirror those reported in prior studies, highlighting persistent systemic gaps in routine hypertension workups. Failure to perform lower limb blood pressure measurements and the initial missed diagnoses via echocardiography reflect real-world deviations from established guidelines and emphasize the need for institutional quality improvements.

The sensitivity of echocardiography for detecting CoA varies significantly depending on the examiner’s expertise and the equipment used. A study reported that the sensitivity of echocardiography was 72.9% at a specialized center, but only 18.5% at a local hospital [7]. These findings underscore the importance of maintaining a high index of suspicion. In our case, two initial echocardiographic evaluations failed to detect CoA. However, a third echocardiogram performed by a pediatric cardiologist ultimately led to the diagnosis. The earlier echocardiograms may have failed to detect the aortic arch abnormality due to limited suprasternal views or lack of pediatric-specific expertise, both of which are known limitations in non-specialized settings.

While echocardiography remains the preferred initial imaging modality due to its accessibility and non-invasiveness, its limitations necessitate consideration of advanced imaging techniques when clinical suspicion persists. CT and MRI are highly effective diagnostic tools, offering superior visualization of the aortic arch and providing critical information for assessing the severity and anatomical extent of CoA. One study reported a 100% detection rate for CoA using these modalities [7]. Thus, when echocardiographic findings are inconclusive, CT and MRI should be considered for further assessment.

This case highlights the need for clinicians to maintain a high index of suspicion for CoA in pediatric patients with treatment-resistant hypertension. A systematic approach - including blood pressure assessment in all four extremities, thorough pulse examination, and appropriate use of imaging modalities - is essential for early detection. In children with persistent or treatment-resistant hypertension, early referral to pediatric cardiology is crucial. Specialist involvement plays a key role in guiding further diagnostic workup, including repeat echocardiography or advanced imaging, to avoid delayed diagnosis and optimize care. This case also underscores the need for systemic improvements in pediatric hypertension evaluation, including consistent adherence to established guidelines and institutional protocols mandating four-limb blood pressure measurement and pulse assessment.

## Conclusions

This case underscores the importance of a structured approach to the evaluation of pediatric hypertension. CoA is a recognized but frequently underdiagnosed cause of secondary hypertension, particularly in younger children, including preadolescents, as in this case. Failure to measure blood pressure in both the upper and lower extremities and to assess femoral pulses can lead to diagnostic delays, despite these steps being recommended in pediatric hypertension guidelines. Although echocardiography is the first-line imaging modality, it may yield false-negative results. When clinical suspicion remains high, repeat echocardiography or advanced imaging such as CT or MRI should be pursued to confirm the diagnosis.
